# Fluorescent biosensors illuminate the spatial regulation of cell signaling across scales

**DOI:** 10.1042/BCJ20220223

**Published:** 2023-10-30

**Authors:** Anne C. Lyons, Sohum Mehta, Jin Zhang

**Affiliations:** 1 Department of Pharmacology, University of California, San Diego, La Jolla, CA 92093, U.S.A.; 2 Shu Chien-Gene Lay Department of Bioengineering, University of California, San Diego, La Jolla, CA 92093, U.S.A.; 3 Department of Chemistry and Biochemistry, University of California, San Diego, La Jolla, CA 92093, U.S.A.; 4 Moores Cancer Center, University of California, San Diego, La Jolla, CA 92093, U.S.A.

**Keywords:** cellular localization, fluorescent biosensors, intracellular signaling, kinases, organelles

## Abstract

As cell signaling research has advanced, it has become clearer that signal transduction has complex spatiotemporal regulation that goes beyond foundational linear transduction models. Several technologies have enabled these discoveries, including fluorescent biosensors designed to report live biochemical signaling events. As genetically encoded and live-cell compatible tools, fluorescent biosensors are well suited to address diverse cell signaling questions across different spatial scales of regulation. In this review, methods of examining spatial signaling regulation and the design of fluorescent biosensors are introduced. Then, recent biosensor developments that illuminate the importance of spatial regulation in cell signaling are highlighted at several scales, including membranes and organelles, molecular assemblies, and cell/tissue heterogeneity. In closing, perspectives on how fluorescent biosensors will continue enhancing cell signaling research are discussed.

## Introduction

**Figure 1.**
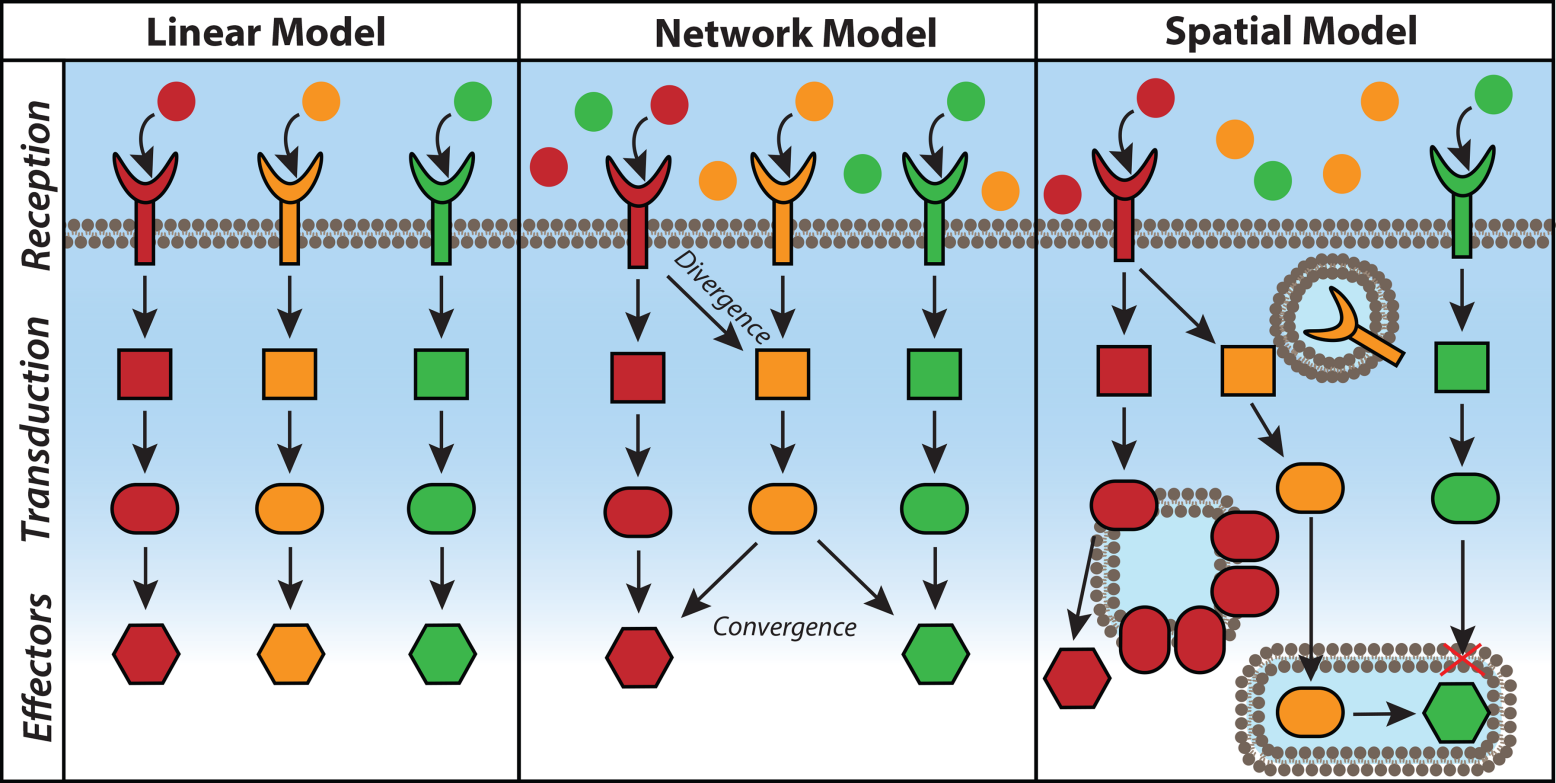
Models of cell signal transduction. (Left) In early signal transduction models, signal reception, transduction, and effector responses were depicted to occur along dedicated, linear pathways in the well-mixed intracellular environment. (Middle) Accumulating knowledge in the cell signaling field has pushed us beyond this linear perspective, revealing that signaling pathways can integrate with each other through divergence and convergence, among other network properties. (Right) Taking spatial localization of signaling elements into account adds additional layers of signaling regulation, as the location and proximity of these elements greatly impact signal transduction.

Cell signaling broadly describes how cells process and transmit information originating from the extracellular or intracellular environment. When the field of cell signaling was initially established, signal transduction was depicted as an essentially linear process: An external signal received at the cell surface is transduced intracellularly through a cascade of second messengers and/or other enzymes that activate effector proteins to drive a cellular response. This view typically assumed that signaling molecules are homogeneously distributed inside cells and that each distinct signal is transduced along a dedicated pathway. These early studies were instrumental in building our knowledge and laying the foundation for our understanding of signaling pathways. However, empirical observations have since demonstrated this initial, simplified model is insufficient to comprehensively describe how signaling pathways achieve specific control of cellular functions, prompting new models of spatially regulated signaling networks [1]. According to this view, signaling molecules are not uniformly present throughout the cell but are heterogeneously distributed at specific subcellular locations, with signal reception and transduction occurring from multiple locations and with the potential for crosstalk with other pathways [2] (Figure 1).

The spatial regulation of cell signaling is exerted at multiple levels. At a subcellular level, signaling pathway activity can vary greatly depending on whether pathway components are located within or on the surface of membrane-bound compartments as opposed to the cytosol. Signaling machinery can also be organized through molecular assembly to form signaling complexes with distinct activities at specific locations in the cell, helping facilitate the spatially confined production or destruction of signaling molecules by local regulatory processes, known as regulatory fencing. Finally, signaling activity can vary among cell populations and across tissues as a result of paracrine and/or endocrine effects. These mechanisms of spatial regulation ensure efficient cellular activities and facilitate acutely controlled signaling with high specificity. Loss of spatial regulation contributes to dysregulated signaling that leads to disease development [2]. Therefore, understanding spatial regulation can critically inform therapeutic development and targeting.

Researchers currently access numerous techniques to unravel the spatial regulation of cell signaling. Traditional biochemical techniques like cell fractionation and Western blotting remain staples of signaling research but involve lysing large numbers of cells to characterize a subset of known molecules, which limits spatiotemporal resolution and can obscure potentially important single-cell behaviors. Multiple ‘omics' technologies are also greatly contributing to our understanding of cell signaling by enabling high-throughput characterization of an array of molecules at discrete time points [3]. Proteomics and phosphoproteomics, for example, are routinely used to characterize protein and phosphoprotein abundance across the whole proteome. The advent of single-cell omics approaches adds further layers of information to explore molecule abundance in contexts of cell and tissue heterogeneity [4,5]. For example, single-cell transcriptomics can identify subpopulations of cells based on mRNA abundance. Even finer spatial detail can be achieved using proximity labeling tools like BioID and turboID, which enable subcellular-scale omics approaches [6–8]. However, fully understanding the spatial regulation of signaling requires tools to monitor dynamic signaling activities with high spatial and temporal resolution within the native biological context of living cells. Genetically encoded fluorescent biosensors offer live-cell compatibility and high spatiotemporal resolution with a modular design that can be adapted to probe numerous signaling pathways. Below, we introduce the basic concepts of biosensor design and application. We then discuss several recent studies that highlight how fluorescent biosensors are being used to illuminate cell signaling across multiple scales of spatial regulation.

## Fluorescent biosensor background

The Nobel Prize-winning discovery and engineering of *Aequorea victoria* green fluorescent protein (GFP) into a rainbow of fluorescent protein (FP) variants transformed the study of cell signaling by enabling the development of genetically encoded fluorescent biosensors [9–11]. The basic job of a fluorescent biosensor is to detect a biochemical input and produce a corresponding optical output. In some cases, specific mutations can directly sensitize an FP to its surrounding environment. However, this approach is generally limited to monitoring pH changes or certain ions (e.g. Cl^−^) [12–16]. A more universal design strategy is using one or more FPs as a ‘reporting unit' along with a separate ‘sensing unit' that detects the biochemical event of interest. The sensing unit typically includes protein or peptide modules, often derived from native cellular components, that take part in the signaling pathway of interest. Many calcium biosensors, for example, include the calcium effector protein calmodulin in the sensing unit [17]. Biosensors for reporting kinase activity typically feature a sensing unit designed to act as a surrogate substrate for the kinase of interest through the inclusion of a kinase-specific phosphorylation sequence and a phospho-amino acid binding domain (PAABD) [18]. The sensing and reporting unit are integrated in specific configurations to allow the detection of diverse biochemical events to modulate the fluorescence readout produced by the coupled FP(s).

Among the simplest sensor configurations are translocation-based biosensors, in which a specific signaling protein or protein domain is fused to an FP and used to indicate the accumulation and/or distribution of a target molecule based on the redistribution of the fluorescence signal from one part of the cell to another. Many phosphoinositide biosensors feature this design, attaching FPs to different lipid-binding domains (e.g. pleckstrin homology domains) [19–21]. Other translocation-based biosensors have also been developed that use the shuttling of the biosensor between two pre-defined locations to report changes in signaling activity, as is the case with kinase translocation reporters (KTRs), whose fluorescence signal moves between the nucleus and cytoplasm depending on the activity of the target kinase [22].

By far the largest and most successful class of biosensors are switch-engineered biosensors. In this design, the sensing unit acts as a molecular switch and undergoes a conformational change upon detection of a biochemical event, which then modulates the fluorescence of the reporting unit. A molecular switch can be directly incorporated into an FP β-barrel, such that conformational changes will distort the FP structure to alter the chromophore environment and, therefore, FP photophysical properties (e.g. intensity, excitation or emission maximum) [23,24]. These sensors are typically engineered using circularly permuted FPs, where the N- and C-termini are relocated within the β-barrel, though other insertion strategies have also been used [24,25].

Such single-fluorophore switch-engineered biosensors are becoming increasingly popular for their high sensitivity and compatibility with multiplexed biosensor imaging [26–28]. However, the most commonly used configuration remains inserting a molecular switch between a pair of FPs that constitute a Förster resonance energy transfer (FRET) pair. FRET is a photophysical process where an excited fluorophore (donor) can non-radiatively transfer its excited-state energy to a second fluorophore (acceptor) [29]. In addition to donor and acceptor photophysical properties (e.g. overlapping spectra between donor emission and acceptor excitation), FRET is dependent on the distance and orientation of the two fluorophores, often being referred to as a molecular ruler [30]. Thus, by dynamically modulating FP proximity, the molecular switch converts the detection of a biochemical event into a FRET change. Commonly used FRET pairs include a cyan-emitting donor and yellow-emitting acceptor (i.e. C/Y FRET) or a green-emitting donor and red-emitting acceptor (i.e. G/R FRET) [31]. Since FRET results in increased emission by the acceptor upon donor excitation, as well as quenching of donor fluorescence and a decrease in the lifetime of the donor-excited state, FRET-based biosensor responses are generally recorded as changes in the acceptor-to-donor emission ratio or in the donor fluorescence lifetime.

Being genetically encodable means that fluorescent biosensors can be constructed using standard molecular biology techniques and introduced to cells as DNA, whereby the cell's own protein synthesis machinery generates the biosensor(s). Genetic encodability also offers the opportunity for enhanced spatial selectivity, as one can harness native cellular machinery to tailor biosensor localization to match the spatial scale of the biological question [32]. For example, subcellular-scale questions can be addressed with great precision by using endogenous localization signals or fusion to a protein of interest, while cell- and tissue-specific promoters can be utilized to address larger-scale questions. However, given the diversity of biosensor configurations, it's important to consider which biosensors are best suited to the biology in question. The often qualitative response of translocation-based biosensors, particularly KTRs, makes them a popular choice for cell- and tissue-scale studies [33–36], whereas switch-engineered biosensors, whose responses are not tied to their localization, are apt for examining subcellular spatial regulation using the above targeting approach. Subcellular targeting can, however, influence biosensor dynamic range — the maximum fluorescence change that can be achieved upon sensor activation — which determines the ability to detect subtle activity changes in smaller signaling pools. Whenever possible, the biosensor with the highest dynamic range should be chosen to maximize sensitivity, and numerous strategies can be employed to increase the sensor dynamic range [37,38].

## Illuminating the scales of spatial regulation

Parallel technological advancements in molecular engineering and microscopy have enabled researchers to go beyond initial theories of linearized cell signaling to directly visualize previously undiscovered aspects of spatial regulation in cell signaling. Within the scope of this review, we highlight recent biosensor developments that reveal the spatial regulation of cell signaling at the level of membranes and organelles, molecular assemblies, or cell/tissue heterogeneity.

## Membranes and organelles

Membrane-bound organelles represent enclosed signaling compartments whose physical boundaries sequester specific biochemical processes while excluding others. The surfaces of lipid membranes can also serve as signaling platforms with distinct properties from those of the bulk cytosolic environment. Biosensors can be directly targeted to different subcellular compartments through the inclusion of sequences encoding endogenous organelle-localization signals or by fusing a biosensor to a protein known to be enriched on or in a given organelle.

### PI3K/Akt/mTOR signaling

Understanding the regulation of metabolic activities across organelles is a natural fit for this approach, as individual organelles engage in distinct catabolic and anabolic processes to respectively break down or build up the intracellular and extracellular environment [39,40]. Anabolic activities are regulated by the protein kinase B (PKB, or Akt) and mechanistic target of rapamycin (mTOR) signaling axis. Akt is a key mediator of the phosphatidylinositol 3-kinase (PI3K) pathway important in oncogenesis and metabolism [41,42]. G-protein coupled receptors (GPCRs) or growth factor receptors are canonically triggered through ligand interaction at the plasma membrane, which activates PI3K to generate phosphatidylinositol-3,4,5-triphosphate (PIP3) to activate Akt. Akt activates mTOR complex 1 (mTORC1) by (1) inhibiting tuberous sclerosis complex 2 (TSC2) to relieve its inhibition of the Ras homolog Rheb, an mTORC1 activator, and (2) inhibiting the proline-rich Akt substrate PRAS40 from binding to the mTORC1 scaffold protein Raptor. mTORC1 is also sensitive to amino acids through Rag GTPases independent of Akt. Beyond the plasma membrane, Akt has been found at numerous subcellular compartments, including lysosomes, the nucleus, and other locations, sometimes showing isoform-specific localization [43,44]. mTORC1 is canonically active at lysosomes [45]. While molecular localization of PI3K pathway components provides vital clues as to where signaling may occur, targeted fluorescent biosensors are being used to directly elucidate the regulation of different activity pools in response to distinct upstream inputs.

#### Lysosomal PI3K

The targeting of phosphoinositide-, Akt-, and mTORC1-specific biosensors has identified specific lysosomal regulation in PI3K signaling. Lysosomes have emerged to be a functionally important site for the Akt–mTORC1 pathway [46,47]. However, how this pathway is regulated at lysosomes is not well understood. Several Akt biosensors, based on both C/Y FRET and single-fluorophore designs, have been developed to monitor Akt activity using an Akt consensus substrate coupled to an forkhead associated 1 (FHA1) PAABD [26,48–50]. To sensitively investigate finite endomembrane PI3K signaling pools, Chen et al. [48] generated an improved single-fluorophore Akt biosensor, called ExRai-AktAR2, obtaining an over 4-fold improvement in dynamic range by mutating the linkers connecting cpEGFP to the Akt substrate and FHA1 domain. This sensor was used to investigate endomembrane Akt activity pools using numerous subcellular targeting motifs, which led to the detection of dynamic Akt activity on the lysosomal membrane.

Having detected Akt on the lysosomal membrane by expansion microscopy, a super-resolution method that involves sample expansion [51], Chen et al. investigated how Akt is recruited to this location by testing the hypothesis that 3-phosphoinsitides (including PIP_3_ and PI(3,4)P_2_) are present on the lysosomal membrane to recruit Akt, using an indicator for phosphoinositides based on Akt (InPAkt). This biosensor contains a molecular switch comprising the pleckstrin homology (PH) domain of Akt linked to a negatively charged pseudoligand peptide and exhibits a C/Y FRET change upon displacement of the pseudoligand by the binding of PIP_3_ or PI(3,4)P_2_ to the PH domain [52]. InPAkt targeted to lysosomes via fusion to the lysosome-enriched glycoprotein lysosome-associated membrane protein 1 (LAMP1) revealed the accumulation of 3-phosphoinositides on the lysosomal membrane in response to growth factor stimulation. Inhibiting dynamin-dependent endocytosis was found to abolish growth factor-induced accumulation of lysosomal 3-phosphoinositides. Under these same conditions, imaging of LAMP1-fused ExRai AktAR2 showed that lysosomal Akt activity was completely abolished, which was corroborated by significantly reduced localization of Akt to lysosomes revealed by expansion microscopy. To examine how the loss of lysosomal Akt activity impacted downstream mTORC1 activity, Chen et al. then used the C/Y-FRET-based mTORC1 activity reporter (TORCAR), which contains the full-length mTORC1 substrate eIF4E binding protein 1 as the sensing unit and shows decreased yellow to cyan emission upon phosphorylation by mTORC1 [53]. Indeed, LAMP1-fused TORCAR also showed little response to growth factor stimulation when dynamin-dependent endocytosis was inhibited. From these results, the authors proposed a model in which 3-phosphoinositides are trafficked to the lysosome surface via dynamin-mediated endocytosis in order to promote growth factor-induced Akt and mTORC1 activity at this location [48]. Overall, the application of fluorescent biosensors to monitor multiple components of the PI3K pathway, namely, PIP3, Akt, and mTORC1, highlights how biosensors can synergistically describe sequential compartmentalized signaling regulation and help identify unique compartment-specific dynamics.

#### Nuclear Akt/mTOR

Beyond lysosomal PI3K signaling, studies using targeted fluorescent biosensors have also identified distinct Akt/mTORC1 regulation in the nucleus. From the earliest observations of Akt and mTORC1 in nuclear fractions of cell lysates, multiple groups have been attracted to the potential of Akt/mTORC1-mediated transcriptional regulation controlling survival, protein synthesis, and the metabolic capacities of the cell, especially considering the hyperactivation of Akt and mTORC1 in some cancers [54–57]. Studies using TORCAR and a C/Y-FRET-based Akt biosensor fused to a nuclear localization sequence (NLS) have confirmed the existence of nuclear pools of Akt and mTORC1 activity [49,53]. However, how these activities are regulated was not understood.

To approach this question, Zhou et al. [58] developed Akt Substrate-based Tandem Occupancy Peptide Sponge (Akt-STOPS) which contains three tandem copies of an Akt substrate peptide to enable targeted inhibition of Akt activity at specific subcellular locations. As illustrated by the responses from a subcellularly targeted, C/Y-FRET-based Akt biosensor, AktAR2, Akt-STOPS was able to functionally inhibit Akt activity at specific locations, and nuclear Akt-STOPS was used to directly investigate whether nuclear mTORC1 was dependent on nuclear Akt activity. Crucially, growth-factor induced nuclear mTORC1 activity was abolished in cells expressing nuclear Akt-STOPS, while amino acid stimulation, which induces mTORC1 activity independent of Akt, was still able to promote nuclear mTORC1 activity. Further mechanistic investigation demonstrated that growth factor-induced nuclear mTORC1 activity depends on the phosphorylation of Ran GTPase binding protein 3 (RanBP3) by nuclear Akt, which facilitates the nuclear import of Raptor to potentiate nuclear mTORC1 activity, as well as Akt-mediated phosphorylation of PRAS40 [58]. A subsequent study using TORCAR to examine nuclear mTORC1 activity identified an additional regulatory mechanism in which a small pool of active Rheb, which is typically directed to associate with membranes via C-terminal farnesylation, instead localizes to the nucleus and plays a critical role in regulating nuclear mTORC1 [59]. While the functional consequences of nuclear Akt and mTORC1 signaling are still being explored, these biosensor studies fortify a time-resolved mechanistic foundation for nuclear Akt and mTORC1 activity regulation and provide powerful tools for continued investigation.

### AMPK signaling

Acting opposite mTORC1-directed anabolic signaling, adenosine monophosphate (AMP)-activated kinase (AMPK) regulates distinct catabolic processes across the cell to promote energy production [60]. AMPK stimulates mitochondrial biogenesis and fission, directs progression and transcriptional control of autophagy, inhibits lipid biosynthesis at the Golgi apparatus and endoplasmic reticulum (ER), and may undergo nuclear shuttling with implications for further transcriptional regulation [61–67]. AMPK is predominantly regulated by two upstream kinases, liver kinase B1 (LKB1) and calcium/calmodulin-dependent protein kinase kinase 2 (CAMKK2), which mediate AMPK responses to energy stress and calcium elevations, respectively, and phosphorylate the AMPKα subunit [60]. AMPK has four binding sites for AMP to detect varying levels of energy depletion, as increased AMP binding conformationally maintains AMPK activation, preventing dephosphorylation [68,69].

Early C/Y-FRET-based AMPK biosensors confirmed that metabolic stress activates AMPK in the cytoplasm and at the plasma membrane, as well as on the Golgi, ER, mitochondrial, and lysosomal surfaces [70,71]. One of these FRET-based AMPK biosensors was used to identify organelle-specific regulation of distinct AMPKα isoforms and demonstrate that selectively inhibiting mitochondrial AMPK activity using a subcellularly targeted AMPK inhibitor peptide decreased intracellular ATP [71]. Importantly, all of these FRET-based AMPK biosensors, which sandwich an FHA1 domain and AMPK substrate peptide between a cyan- and yellow-emitting FP pair exhibit modest dynamic ranges, thus hampering detailed kinetic assessments of AMPK activation. Given the diverse upstream regulation and downstream metabolic effects of AMPK activity, characterizing AMPK regulation at varying subcellular locations is crucial for informed drug design for cancer and metabolic diseases.

#### Nuclear, lysosomal, and mitochondrial AMPK

**Figure 2.**
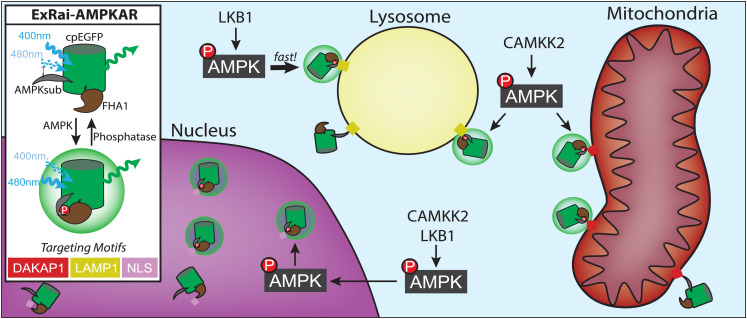
Organelle-level AMPK activity at nucleus, lysosomes, and mitochondria. The excitation ratiometric AMPK activity reporter (ExRai AMPKAR) senses AMPK activity using a molecular switch composed of an FHA1 domain and an AMPK substrate, which undergoes a conformational change upon phosphorylation. This conformational change shifts the maximum excitation wavelength of the cpEGFP reporting unit from 400 nm to 480 nm, which is quantified as the ratio of fluorescence intensities at these two wavelengths. Fusing targeting motifs to ExRai AMPKAR enables analysis of AMPK activity at subcellular compartment under various perturbations, including knockout of upstream kinases to identify whether LKB1 or CAMKK2 regulate compartment-specific signaling.

Indeed, recent work targeting an improved AMPK biosensor to various organelles has revealed distinct compartment-specific signaling kinetics, including isoform-specific and upstream-kinase-dependent effects. Using the sensing components found in previous C/Y-FRET AMPK biosensors, Schmitt et al. [72] were able to generate a single-fluorophore AMPK reporter with dramatically improved sensitivity. By sandwiching cpEGFP between the AMPK substrate and FHA1 domain, the authors obtained a sensor whose phosphorylation-induced conformational change yielded a 3-fold higher dynamic range in response to AMPK activity versus earlier C/Y-FRET-based designs (Figure 2). This improved reporter, named ExRai AMPKAR, had sufficient dynamic range to sensitively visualize compartment-specific signaling kinetics. In addition to nuclear targeting with an NLS and lysosomal targeting with LAMP1, ExRai AMPKAR was also targeted to the outer mitochondrial membrane using a fragment of dual-specificity A-kinase anchoring protein 1 (DAKAP1).

While previous investigations did not show meaningful nuclear AMPK activation in response to metabolic stress, ExRai AMPKAR facilitated robust detection of LKB1-induced nuclear AMPK activity in response to metabolic stress, as well as through direct allosteric activation. To clarify the regulation of nuclear AMPK activity, the ability of nuclear-localized or diffusible AMPKα2 to promote nuclear AMPK activity in AMPKα knockout cells was compared, demonstrating that only diffusible AMPKα2 was able to drive nuclear AMPK activity. These results helped formulate a mechanistic model of nuclear AMPK activity in which AMPK is first activated in the cytosol and then translocates into the nucleus to phosphorylate downstream targets. This model and associated methods of studying nuclear AMPK translocation and activity are particularly useful, as downstream AMPK targets that are exclusively nuclear, rather than fellow shuttling components, are under active investigation [61]. Applying ExRai AMPKAR in upstream kinase knockout conditions can also identify distinct upstream inputs required for specific subcellular pools of AMPK activity. In comparing lysosomal, mitochondrial, and cytosolic AMPK dynamics, LKB1 was found to be critical for driving maximal AMPK activity in the cytosol and rapid AMPK activation at lysosomes, but was largely dispensable for mitochondrial AMPK activation. Conversely, CAMKK2 appeared critical for maximal AMPK activity across all three locations. Identifying these differential roles of upstream kinases at different subcellular locations offers potential pharmacological insights into activating specific pools of AMPK [73]. Overall, sustained efforts to improve the utility of AMPK biosensors continue to yield novel biological insights in its control of catabolic signaling with distinct spatiotemporal regulation.

### GPCR-mediated ERK signaling

Carefully executed organelle- and membrane-targeting approaches can even upend established signaling paradigms, as is the case with a recent study of GPCR-mediated activation of extracellular regulated kinase (ERK) signaling. As GPCRs are an abundant family of signaling receptors targeted by over a third of existing small-molecule drugs, it is critical to understand the molecular mechanisms of GPCR signal transduction [74]. As their name suggests, these receptors are coupled to different heterotrimeric G proteins, where the Gαs isoform is coupled to cyclic adenosine monophosphate (cAMP)-dependent signaling. In addition to signaling through their coupled heterotrimeric G proteins, another major downstream target of GPCR signaling is the ERK pathway, which broadly regulates cell proliferation, differentiation, and apoptosis. Canonically, GPCR-stimulated ERK pathway activation is thought to be mediated by β-arrestins, which, alongside their key role in facilitating GPCR endocytosis, have been shown to act as scaffolds for ERK pathway components [75–79]. However, this classical model has become controversial in light of recent studies suggesting that β-arrestins do not play a role in GPCR-stimulated ERK signaling [80,81]. Furthermore, a growing body of work has revealed that, far from attenuating signaling, ligand-induced GPCR endocytosis produces a spatially distinct intracellular GPCR signaling compartment with potentially unique signaling functions [82]. The role of internalized GPCRs in regulating ERK signaling has thus far been unclear.

#### Endosomal GPCR-mediated ERK signaling

Using the well-studied β2 adrenergic receptor (β2AR) as a model, Kwon et al. [83] sought to clarify the spatial regulation of GPCR-induced ERK signaling, and the role of β-arrestins, by targeting an ERK biosensor to different subcellular compartments. Specifically, they selected the C/Y-FRET-based biosensor EKAR4, containing an ERK substrate and phospho-amino acid binding WW domain tethered by an EV linker to improve dynamic range [84], which they targeted to either the plasma membrane using a KRAS tag or early endosomes using tandem FYVE motifs [85]. Surprisingly, plasma membrane-targeted EKAR4 did not detect the expected ERK activity at the plasma membrane following β2AR stimulation, even after accounting for potential inhibitory PKA crosstalk or when forcing β2AR to remain at the plasma membrane. EKAR4 targeted to endosomes, however, showed a robust ERK response to β2AR stimulation. This β2AR-stimulated endosomal ERK activity was disrupted by endocytosis inhibition, as well as by genetic knock-down of β-arrestin or knock-out of the Gαs G-protein subunit. The authors also utilized a suite of molecular tools to perturb the β2AR signaling machinery, including nanobodies, which are single-chain antibody fragments that can be genetically encoded. Three molecular tools for β2AR perturbation were utilized: nanobody Nb80, which binds active β2AR and blocks signaling to Gαs, nanobody Nb37, which stabilizes the active, open conformation of Gαs and promotes signaling, and GsCT, a peptide inhibitor of Gαs [86–88].

By targeting these molecular tools to either endosomes or the nucleus, the authors sought to manipulate β2AR signaling in a location-specific manner. Responses from endosome- and nuclear (NLS)-targeted EKAR4 demonstrated that active, endosome-localized β2AR signaling machinery is required for ERK activity at both endosomes and the nucleus. In particular, biochemical experiments suggested that active, endosome-localized Gαs directly recruits the upstream components RAF1 and MEK1 to initiate endosomal ERK activity. These results delivered a new model of non-canonical GPCR-mediated ERK signaling where β-arrestin facilitates GPCR endocytosis and endosomally localized active Gαs recruits RAF1 and MEK1 to activate ERK signaling, which ultimately propagates to the nucleus. This model showed further promise when considered in the context of myelodysplastic syndrome, which describes a group of blood cancers typified by a long splice variant of Gαs and up-regulated ERK activity [89]; indeed, Kwon et al. found that this long Gαs splice variant was exclusively associated with β2AR-induced endosomal and nuclear ERK activity, in contrast with the short Gαs isoform. Informed by the use of membrane- and organelle-targeted fluorescent biosensors, this new model has the potential to significantly impact the GPCR drug development landscape, especially for numerous cancers featuring hyperactive and/or aberrant overexpression of GPCRs [90].

## Molecular assemblies

Molecular assemblies represent micro- or nanometer-scale signaling compartments that are organized directly at the protein level. In particular, the ability of many signaling proteins to incorporate into so-called ‘membraneless organelles' has attracted intense interest in the last decade, spurring investigations into their role in the spatiotemporal regulation of intracellular signaling. These dynamic assemblies, also referred to as biomolecular condensates, form through a process known as liquid–liquid phase separation (LLPS), which describes a phenomenon whereby groups of molecules overcome the tendency to be homogenously distributed in solution and spontaneously coalesce into membraneless, liquid-like droplets that are physically distinct from the surrounding environment [91].

### PKA/cAMP signaling

Recently, work by Zhang et al. [91] revealed that the α isoform of the type I regulatory (R) subunit (RIα) of cAMP-dependent protein kinase (PKA) is capable of undergoing LLPS to form liquid-like condensates both *in vitro* and in living cells. Notably, the authors took advantage of the spontaneous fragment complementation of split superfolder GFP (sfGFP) to examine the behavior of endogenously expressed RIα. In this system, the eleventh β-strand of sfGFP (sfGFP_11_) and the remaining ten β-strands (sfGFP_1-10_) are expressed as separate fragments that show no fluorescence alone but spontaneously assemble to reconstitute fluorescent sfGFP when co-expressed [92]. The small size of sfGFP_11_ facilitates its insertion into endogenous genomic loci using CRISPR/Cas9 technology, so that sfGFP_1–10_ can be reconstituted at an endogenously expressed protein of interest (POI) [93], in this case PKA RIα. Using this approach, Zhang et al. were able to demonstrate not only that RIα phase separates in cells but that it does so at endogenous expression levels.

#### Endogenous PKA/cAMP in phase separation

A tetrameric holoenzyme consisting of an R subunit dimer bound to a pair of catalytic (C) subunits, PKA becomes activated when cAMP binds the R subunits and triggers an allosteric switch that unleashes PKA-C to phosphorylate downstream targets. Notably, Zhang et al. [94] observed that co-incubation with purified PKA-C actually suppressed RIα LLPS *in vitro,* but that this could be reversed by adding cAMP, indicating that cAMP regulates RIα LLPS. Indeed, they also found that treatment with cAMP-elevating stimuli rapidly up-regulated the formation of RIα condensates in cells. Finally, when the authors co-expressed FP-tagged RIα and PKA-C in cells, they observed PKA-C to co-phase separate with RIα, prompting them to examine cAMP and PKA dynamics directly within these phase-separated bodies [94].

To investigate signaling dynamics associated with molecular-scale assemblies, it is important to engineer biosensors that can probe signaling activities at a comparable spatial scale. For example, analogous to how biosensors can be targeted to organelle membranes via fusion to localization motifs, biosensors are often fused to POIs to visualize nearby signaling. Resolving signaling activities at specific molecules, however, raises concerns about the biological limitations of overexpression systems [95]. In particular, overexpressing a POI-fused biosensor risks perturbing native signaling dynamics by altering the endogenous stoichiometry of the targeted molecular assembly [96]. Nanobody targeting of biosensors to a POI is one potential solution, but developing a suite of highly specific nanobodies is not trivial [97]. Furthermore, responses from excess, mislocalized biosensor may confound analysis of local signaling. To address this, Zhang et al. [94] devised a novel biosensor complementation strategy by engineering fluorescent sensors targeted to endogenous proteins (FluoSTEPs). As above, this approach relies on split sfGFP, but instead of simply recruiting sfGFP_1–10_ to a POI endogenously tagged with sfGFP_11_, FluoSTEPs pair sfGFP_1-10_ as a partial FRET donor with a red-emitting FRET acceptor, such that reconstitution of sfGFP produces a functional G/R FRET biosensor only at the location of the endogenously expressed POI, eliminating issues related to POI overexpression.

**Figure 3.**
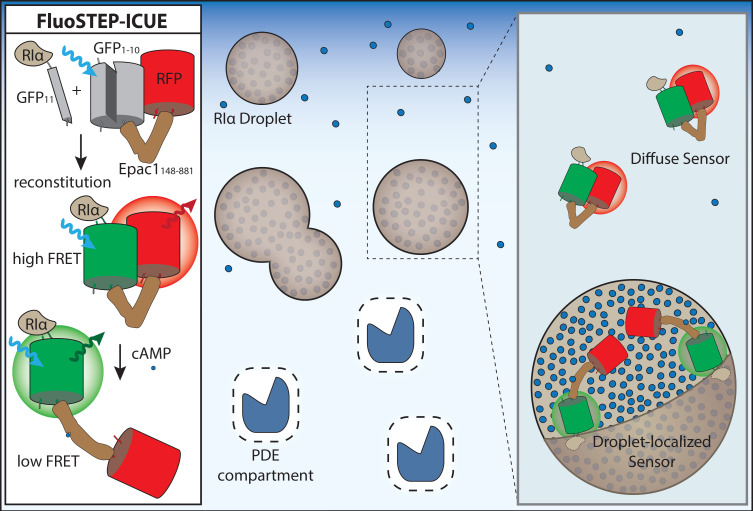
Microdomain-level PKA and cAMP in phase separation. The fluorescent sensors targeted to endogenous proteins (FluoSTEP) approach with indicator of cAMP using Epac1 (ICUE) was used to investigate cAMP dynamics near endogenously expressed PKA RIα. The high levels of cAMP present within RIα phase-separated condensates result in cAMP binding to the Epac1 domain of ICUE, leading the sensor to adopt a conformation in which FRET between GFP and RFP is reduced. Outside of RIα condensates, where cAMP concentrations are far lower in the basal state, ICUE is not bound to cAMP and adopts a conformation that results in higher FRET between GFP and RFP.

FluoSTEPs closely mirror the designs of existing FRET-based biosensors and can be readily generalized to detect various signaling targets [96]. Specifically, Zhang and colleagues utilized FluoSTEP versions of A-kinase activity reporter (FluoSTEP-AKAR), in which a PKA substrate tethered to an FHA1 domain serves as the sensing unit to detect PKA activity, and indicator of cAMP using Epac (FluoSTEP-ICUE), which utilizes the intrinsic cAMP-induced conformational change in the cAMP effector Epac1 as the sensing unit (Figure 3). By reconstituting FluoSTEP-AKAR and FluoSTEP-ICUE at endogenously tagged RIα, Zhang et al. [94] were able to reveal that RIα condensates exhibited high levels of cAMP accumulation and PKA activity compared with the diffuse pool of RIα. New RIα condensates that formed in response to cAMP stimulation were further shown to exhibit stronger FluoSTEP-AKAR and FluoSTEP-ICUE FRET responses than diffuse RIα regions, suggesting that these phase-separated bodies become dynamically enriched in cAMP and active PKA. Given these behaviors, the authors then speculated whether RIα condensates play a role in regulating cAMP compartmentation.

#### cAMP regulatory fencing and compartmentation

cAMP signaling is generally initiated at the plasma membrane following ligand binding to Gαs-coupled GPCRs, which activate adenylate cyclases (ACs) to produce cAMP from ATP. cAMP then binds various downstream effectors, including PKA, which regulates cell proliferation, differentiation, and multiple tissue-specific processes [98]. With hundreds of GPCRs converging onto cAMP in response to diverse extracellular ligands [99], the mechanistic challenge faced by cells to ensure specificity in cAMP/PKA signaling cannot be understated. One way cells are thought to handle this problem is through cAMP compartmentation, whereby cAMP elevations do not spread uniformly throughout the cell but are instead confined to discrete locations [100]. cAMP-degrading phosphodiesterases (PDEs), which attenuate cAMP signaling and serve as the functional counterpart to ACs, are considered principal mediators of cAMP compartmentation by locally degrading cAMP and thus establishing boundaries, or regulatory ‘fences', that restrict cAMP diffusion into or out of signaling compartments. Indeed, cAMP compartmentation has been observed through the formation of diffusible gradients or microdomains in several contexts [101,102].

However, this model of PDE-driven compartmentation is contradicted by computational studies based on early observations that cAMP diffusion is unrestricted in cells [103–105]. Fluorescent biosensors are uniquely suited to probe this question, and in a recent study, Bock et al. [106] employed FRET-based ‘nanorulers', in which PDE isoforms were joined by rigid alpha-helical linkers to the C/Y-FRET-based Epac1 cAMP sensor (Epac1-camps) or the PKA sensor AKAR4, to investigate cAMP accumulation and PKA activity within a defined radius surrounding individual PDE molecules. These studies showed that PDEs easily degrade cAMP and block PKA activation within a nanoscale domain under low cAMP conditions. These results were supported by microscopic analyses indicating that cAMP diffuses more slowly in cells than previously estimated. To account for the slow cAMP diffusion they observed, the authors proposed a model in which cAMP is bound to intracellular buffers under physiological conditions to enable the formation of PDE nanodomains that compartmentalize cAMP.

Intriguingly, the cAMP- and PKA-enriching behavior of RIα biomolecular condensates suggested that they may in fact function as a cAMP buffering system. To investigate the possible interplay between PDEs and RIα condensates, Zhang et al. [94] modified the above cAMP nanoruler approach by fusing the C/Y-FRET-based cAMP sensor ICUE4 to a PDE catalytic domain to directly monitor cAMP compartmentation in the context of RIα condensate formation and disruption. Strikingly, when RIα condensate formation was present, minimal cAMP was observed to accumulate near PDEs, whereas disrupting condensate formation led to robust cAMP accumulation around PDEs. Furthermore, the little cAMP that accumulated within the PDE nanodomain in the presence of RIα condensates was totally abolished when RIα was overexpressed, indicating that RIα condensates support the formation of PDE-driven cAMP nanodomains by functioning as the predicted cAMP buffers, producing a sophisticated cAMP compartmentation system. The pathophysiological implications of this novel finding were further demonstrated by the discovery that a PKA-C fusion oncoprotein found in an atypical liver cancer [107] potently disrupts RIα condensate formation and cAMP compartmentation in cells, inducing tumorigenic phenotypes [94].

#### GPCR specificity in cAMP compartmentation

The cAMP-nanoruler approach pioneered by Bock et al. has also recently proven instrumental in deciphering another key question in cAMP signaling, namely, how cells are able to distinguish cAMP elevations induced by specific GPCRs. To investigate this question, Anton et al. [108] fused Epac1-camps to a pair of GPCRs, specifically, the glucagon-like peptide-1 receptor (GLP-1R) and β2AR, via rigid alpha-helical linkers of different lengths. Similar to the investigation of PDE nanodomains, these rigid linkers acted as rulers to measure cAMP gradients along the plasma membrane within nanometer-scale domains surrounding these receptors. The authors compared the responses of these GPCR-fused nanoruler sensors with those from Epac1-camps localized to the general plasma membrane, via KRAS tagging, or within the bulk cytosol. Using this approach, Anton and colleagues were able to reveal the formation of receptor-associated independent cAMP nanodomains (RAINs), whereby spatially confined cAMP gradients form locally around individual GPCRs upon agonist stimulation. Specifically, application of very low (i.e. physiological) agonist concentrations led to significantly higher cAMP accumulations within RAINs, as indicated using cAMP nanorulers, versus the bulk cytosol, while this difference was eliminated at higher agonist concentrations. Using a similar approach with a tethered version of the PKA activity biosensor AKAR4 [109], the authors showed that cAMP was sufficient to activate PKA within a 60 nm region surrounding the receptor. Furthermore, PDE inhibition extended the radius of the RAIN regions, indicating that PDEs help establish the boundaries of these nanodomains and once again highlighting the importance of regulatory fencing in cAMP compartmentation. Overall, fluorescent biosensors resolved long-standing mechanistic questions regarding the molecular-scale regulation of cAMP/PKA signaling, showing that cAMP is dynamically regulated by multiple levels of molecular assemblies and regulatory fences throughout the cell to achieve specificity towards downstream targets.

#### AKAPs: PKA signaling islands

In addition to cAMP compartmentation, the specificity of PKA signaling is also increased through association with A-kinase anchoring proteins (AKAPs), a group of scaffold proteins that recruit PKA into discrete molecular complexes containing numerous other signaling molecules which regulate distinct functions at varying subcellular locations [110,111]. Over sixty AKAPs have been identified in the human genome to date [112,113], each of which could potentially assemble a uniquely regulated PKA signalosome. However, given the fact that AKAPs interact with PKA holoenzyme via the R subunits and that PKA R and C subunits are canonically thought to dissociate upon cAMP binding, whether AKAPs can spatially confine PKA at all has become somewhat controversial. Direct visualization of PKA activity compartmentation by AKAPs raises questions about what biosensor and optical modality is suited to monitoring such minute, specific molecular-scale activities. Standard epifluorescence microscopy is limited to resolving objects no smaller than half the illumination wavelength, typically ∼250 nm, which is known as the diffraction limit. While this limitation is very reasonable for cellular-scale imaging, it is multiple orders of magnitude away from molecular-scale observations [114–116].

Fortunately, there are now numerous super-resolution microscopy methods that push fluorescence imaging beyond the diffraction limit. For example, photochromic stochastic optical fluctuation imaging (pcSOFI) utilizes reversibly photoswitchable FPs, whose spontaneous flickering emission behavior is used to generate images with a resolution below the diffraction limit [117]. However, these methods have typically lacked the corresponding molecular toolkit to provide information on dynamic signaling activities at the super-resolution level [118]. To address this gap, Mo et al. [119] generated the first super-resolution-compatible activity biosensors by using the reversibly photoswitchable green FP Dronpa to modulate the flickering behavior of the red-emitting FP TagRFP-T, a phenomenon termed fluorescence fluctuation increase by contact (FLINC). FLINC is driven by a physical interaction between TagRFP-T and surface residues on the Dronpa β-barrel and, like FRET, is highly sensitive to the distance between these two FPs. By separating this FLINC FP pair with a PKA substrate, EV linker, and FHA1 domain, the authors generated FLINC-based A kinase activity reporter (FLINC-AKAR1) which, when visualized via pcSOFI, allowed them to reveal the existence of discrete PKA activity domains, roughly 250 nm in diameter, spread across the plasma membrane. Additional experiments performed using another super-resolution technique, stochastic optical reconstruction microscopy [120], to image antibody-labeled fixed cells, revealed that these highly active plasma membrane PKA nanodomains colocalize with clusters of the well-studied PKA scaffold AKAP79. These results demonstrated that AKAPs can indeed organize PKA activity into spatially distinct nanodomains and further informed a release/recapture model in which high concentrations of R subunits within AKAP clusters prevent released PKA-C from diffusing out of these nanodomains. This work advanced the growing perspective that signaling activities are tightly coordinated at even the smallest spatial scales, prompting the development and validation of additional FLINC-based biosensors to monitor protein–protein interactions or other kinase activities, such as ERK.

With the limited options for FPs that can be utilized for super-resolution applications and the Dronpa chromophore being non-critical for FLINC, Lin et al. [121] subsequently developed Dronpa chromophore-removed FLINC (DrFLINC), in which the sensor pairs TagRFP-T with a non-fluorescent Dronpa β-barrel. DrFLINC thus frees up the green emission channel previously occupied by Dronpa, such that Dronpa can now be used to tag additional POIs. This strategy allowed a live-cell demonstration of AKAP79 co-clustering with PKA activity nanodomains using DrFLINC-AKAR1. If implemented in other signaling pathways, FLINC-based sensors could detect flux at the most fundamental spatial level of signaling on the nanodomain level.

## Cell/tissue heterogeneity

While the previous sections focused on subcellular spatial regulation of signaling activity, we now venture into cell and tissue heterogeneity, which encapsulates variation in signaling across gap junctions as well as through paracrine and endocrine effects. Investigating this scale of signaling is critical for understanding how multi-cellular organisms function as different populations of cells execute tasks and signaling activities they are phenotypically specialized to perform. This is where signaling heterogeneity arises, as a specific group of cells may initiate or direct signaling among a larger population or within a specific physicochemical environment. Fluorescent biosensors have utility at this scale to extract information about heterogeneity in cell signaling across time, whereas alternative methods can obscure cell–cell variability or lose temporal information. If observation is only possible at a few discrete time points, the selected frequency and length of observations can hinder the observation of complex temporal behaviors.

### Cell cycle tracking

Cell-to-cell heterogeneity observed in various processes may be tied to cell cycle stage variation, which affects a variety of signaling activities that cells can execute [122–124]. The impact of the cell cycle on cellular processes emphasizes the need for methods to monitor the cell cycle on the single-cell level. To meet this challenge, Sakaue-Sawano et al. [125,126] fused ubiquitin E3 ligase substrates that are differentially expressed and degraded at varying stages of the cell cycle to spectrally distinct FPs to make a series of fluorescent ubiquitin cell cycle indicators (FUCCI). Observationally, this means single cells show color changes and color redistribution depending on their cell cycle stage. FUCCI4 maximally discriminates between growth stage 1 (G1), DNA synthesis (S), growth stage 2 (G2), and mitosis (M), all four stages of the eukaryotic cell cycle, whereas recent FUCCI versions reduce the number of observable stages for *in vivo* cell cycle investigations by using red-shifted FPs [127–129].

Since the first FUCCI was reported, these fluorescent biosensors have broadly impacted both fundamental biology and translational research, particularly in stem cell and cancer research. In studies examining the effect of cell cycle stage on cell fate during embryonic stem cell differentiation, FUCCI has been used to reveal that shorter G1 length is maintained in pluripotency, with cells differentiated in early G1 showing an endoderm fate and cells differentiated in late G1 having a neuroectoderm fate [130–133]. This work has prompted further tissue-specific *in vitro* and *in vivo* investigations to probe connections between cell cycle status and neural and cardiac development in embryogenesis [134–136]. Applying cell cycle knowledge in regenerative medicine, where many aim to increase the proliferative output of cells with therapeutic potential, researchers have used FUCCI to identify methods or drugs that maximize cell yields relative to cell cycle state [137,138]. In using FUCCI to examine the spatial heterogeneity of solid tumors, Yano et al. [139] found that tumor cores contained quiescent G0/G1 cells, while cells near blood vessels or the tumor surface are in S/G2/M, prompting the authors to hypothesize that resistance to cytotoxic therapies is influenced by mitotic activity. In cancer therapeutic screening, FUCCI has been used to reveal methods or drugs that minimize proliferation, while verifying whether cell cycle status affects efficacy [140–143]. Serving yet an even broader purpose, the spatiotemporal resolution of FUCCI sensor data has facilitated the construction and verification of computational models of proliferation, cell cycle arrest, and wound healing [144–146]. The impressive findings yielded by cell cycle reporters emphasize the importance of monitoring cell cycle status.

FUCCI has also proven essential for directly providing new insights into mechanisms of cell cycle regulation. Proliferating cells deprived of mitogenic stimuli are typically observed to form two cell populations: one group of cells that stops dividing and another that continues through the cell cycle [147]. The latter group are said to have irreversibly committed to dividing, having passed through a so-called cell cycle restriction point. At the molecular level, the restriction point is driven by the activation of cyclin-dependent kinases 4 and 6 (CDK4/6) in response to mitogen signaling, leading to phosphorylation of the retinoblastoma (Rb) protein. Phosphorylated Rb induces the expression of cyclins E and A, which then activate CDK2 to trigger additional Rb phosphorylation. The resulting positive feedback loop decouples CDK2 activity from upstream signaling, yielding a bistable system in which cells either exit or commit to the cell cycle depending on a CDK2 activity threshold [148,149]. Canonically, the restriction point is thought to occur in G1, with cells that have passed G1 committing irreversibly to performing another cell cycle regardless of mitogen signaling, whereas cells still in G1 will enter a quiescent, G0 state [150–153]. However, numerous groups have noted outlier cell populations [150,153,154], suggesting that the current model is incomplete.

To investigate this observed heterogeneity in the apparent timing of the restriction point, Cornwell et al. [155] recently performed multiplexed imaging of the G1-specific FUCCI sensor, a CDK1/2 kinase translocation reporter, and a histone marker in cell tracking experiments to discriminate between critical restriction point phases during the cell cycle. By investigating post-restriction point outlier cells in detail, they found that up to 15% of G0 cells had elevated ploidy and low Rb phosphorylation, consistent with cell cycle exit during G2, thus contradicting the model of a CDK2-driving positive feedback loop leading to post-G1 cell cycle commitment. In contrast with a single restriction point decision in G1, the authors postulated a temporal competition model between distinct molecular clocks that drive either cell cycle exit or mitosis. Indeed, by gradually extending the time cells spent in interphase, the authors observed proportional increases in the percent of cells that were fated to exit the cell cycle into G0. Conversely, mitosis-fated cells were shown to require sustained CDK4/6 activity during interphase to stimulate cyclin A2 synthesis, where sufficient cyclin A2 half-life sustains CDK2 activity through mitosis. Thus, the use of fluorescent biosensors revealed a new mechanism of restriction point cell cycle regulation, where mitogens and CDK4/6 are required to maintain CDK2 activity throughout interphase, as opposed to G1 alone, for cells to reach mitosis. More broadly, this study highlights the utility of biosensors for describing time-dependent cell fates that require live, time-resolved observation methods.

### Metabolic heterogeneity

In connection with cell cycle heterogeneity, it is also unreasonable to assume metabolic synchronization among a cell population similarly facing chemical and spatial limitations on growth and survival. While there are long-standing standardized benchmarks for organism-level metabolic homeostasis, describing the underlying contributions of specific tissues or even individual cells is critical for understanding metabolic disorders and developing effective drugs [73,156]. With this interest, we can refer again to the representative example of the low-energy-sensing kinase AMPK, the master regulator of catabolism. In addition to the diverse subcellular roles of AMPK signaling discussed previously, characterizing the tissue-specific roles of AMPK signaling is also heavily pursued as it relates to treating cardiovascular disease, liver disease, and other diseases with apparent tissue-specific metabolic origins [157,158]. Indeed, transgenic mice expressing a C/Y-FRET-based AMPK biosensor show preferential activation of AMPK in skeletal muscle with the AMP analogs AICAR or in liver with metformin, the popular type 2 diabetes drug [159].

#### AMPK & drivers of single-cell metabolic status

While our understanding of tissue-specific metabolic functions grows with AMPK and other pathways, less is known about single-cell metabolic variations. Towards this goal, fluorescent biosensors can be used to directly probe metabolites and signal transduction elements in real time to quantify metabolic heterogeneity. In a recent study from Hung et al. [160] energetic stress from inhibition of glycolysis or oxidative phosphorylation can be monitored through the activity of AMPK as a low-energy sensor, while opposing biosynthetic pathway activity, such as through Akt, can also be monitored. In epithelial cells expressing a C/Y-FRET-based AMPK activity reporter (AMPKAR2) treated with various metabolic inhibitors over several hours, single cells showed distinct AMPK activities that stably increased or dynamically fluctuated, showing pulses and even oscillations, in a drug- and dose-dependent manner. A glyceraldehyde 3-phosphate dehydrogenase (GAPDH) inhibitor called iodoacetate garnered substantial interest, as this induced the greatest frequency of AMPK oscillations, a behavior otherwise obscured by taking the cell population average. Upon parallel investigations through multiplexed imaging of an Akt-KTR with AMPKAR2 or a ratiometric NADH biosensor, AMPK oscillations were found to be coordinated with Akt and NADH oscillations, demonstrating a coordinated balance between catabolism and anabolism. Further multiplexed imaging of AMPKAR2 and FUCCI revealed a connection between dynamic metabolic flux response and cell cycle arrest or even cell death, whereas stable AMPK activation showed no effect on mitotic activities. Using PI3K/Akt inhibitors, the authors demonstrated that Akt plays a crucial role in suppressing fluctuations in energetic stress. While this work illuminated the dynamics of metabolic homeostasis at the single-cell level, questions remained about what distinguishes individual cell metabolic flux behaviors.

Further work using common cell lines expressing AMPKAR2 treated with various oxidative phosphorylation (OP) inhibitors over several hours identified a population of OP-independent cells with little evidence of energy stress and a population of OP-dependent cells with strong AMPK fluctuations [161]. Using additional C/Y-FRET-based biosensors for ATP or ADP/ATP ratio, the authors confirmed that these AMPK activity behaviors represented energetic adaptations to OP inhibition as individual cells attempted to maintain ATP levels. By complementing their investigation with a FUCCI probe, Kosaisawe et al. revealed the contribution of cell cycle phase, as OP-independent cells were enriched in G1 phase, while OP-dependent cells were enriched in S or G2 phase, though both subpopulations had cells at all stages of the cell cycle. Tracking AMPK activity among dividing cells, they also found that OP-dependent and OP-independent behaviors could be transiently inherited by daughter cells. Increasing glucose transporter expression and reducing protein synthesis decreased the observed OP-dependent cell responses. Through the use of fluorescent biosensors, these studies revealed flux-balancing metabolic homeostasis occurring at the single-cell level and that subpopulations of cells have distinct metabolic stress responses depending on glycolytic capacity, cell cycle phase, and protein synthesis capacity. A critical step moving forward will be identifying how cell-level heterogeneity integrates with tissue and organism-level metabolism in health and disease.

### Growth factor-mediated ERK signaling

While we previously discussed GPCR-mediated ERK activity, ERK is primarily activated in response to growth factor signaling, an active area of interest in cancer research as tumors hijack growth factor signaling pathways to fuel their rapid growth [162,163]. Initial studies of growth factor-stimulated ERK activation had time-scale discrepancies attributed to potential pulsatile behavior — while cells were synchronously activated by acute growth factor stimulation, immunofluorescence performed hours later showed signaling separation with distinct populations of ERK-active and inactive cells [164–166]. Identifying how the cells start with a synchronous, acute response and lose synchrony over several hours requires methods with sufficient temporal resolution to work within both short and extended timeframes.

#### ERK subpopulations in proliferation and apoptosis

Fortunately, fluorescent biosensors can meet the temporal need to probe ERK cell–cell signaling heterogeneity across the entire timeframe of interest to investigate when and how cells have variable growth factor-stimulated ERK activity. Based on imaging of a C/Y-FRET based ERK activity reporter with an EV linker (EKARev) over several hours, the signaling heterogeneity was first described as ERK pulses that were frequency modulated by growth factor receptor activity and amplitude modulated by the upstream kinase MEK [37,166,167]. In describing pulsatile signaling, frequency modulation describes changes in pulse rate, while amplitude modulation describes changes in pulse magnitude [168]. Through parallel application of high-content immunofluorescence and FUCCI imaging, Albeck et al. [166] clarified that the previously observed population heterogeneity correlated with the fraction of time individual cells spent with ERK activated and quantified a threshold of ERK activity that would preferentially stimulate cell proliferation. Through an *in vivo* investigation of ERK activity by Hiratsuka et al. [169] this heterogeneity was described as spatial radial Erk activity distributions, or SPREADs, where firework-like bursts of ERK activity visualized with nuclear localized EKARev-NLS were observed at the epidermis of mice and increased by mitogenic stimulus [170]. In the context of wound healing, recurring ERK activity waves were observed parallel to the wound shape. Additional application of FUCCI-expressing mice by Hiratsuka et al. allowed the correlation between SPREADs and the G2/M cell cycle phase to be identified.

As several groups have continued working in this area, more factors that contribute to growth factor-based ERK signaling heterogeneity have been identified, including cell density, growth factor specificity, and further downstream kinases [171–174]. While studies have largely focused on ERK in the context of proliferation, apoptosis is another important context, particularly when considering healthy or aberrant turnover of cells in disease. Using ERK and Akt KTRs, Gagliardi et al. [34] demonstrated that apoptotic cells initiate ERK and Akt activity waves that are transduced as survival signals to the nearest neighboring cells, ensuring the cells closest to an apoptotic site live long enough to maintain epithelial barrier integrity.

#### ERK subpopulations in cell migration

Recent investigations of ERK heterogeneity have been focused on understanding the integration of biochemical and mechanical cues in collective cell migration, critical for understanding embryonic development, cancer cell invasion, and wound healing [36,175,176]. In addition to cell–cell interactions, a variety of environmental cues such as matrix or substrate stiffness and growth factor presence contribute to the polarization of migrating cells into two distinct populations: cells leading the migration front and those following [177]. This polarity is architecturally built using cytoskeletal components helping leader cells reach forward into free space with lamellipodia while retracting their opposite side. While ERK is known to drive numerous cytoskeletal actions through myosin light chain kinase and F-actin polymerization, the connection between ERK activity waves and migration was less clear [178]. By combining EKARev-NLS FRET imaging with traction force microscopy, Aoki et al. [179] found that ERK activity waves precede myosin light chain movement, inducing traction force generation in the opposite direction of the waves. While this work demonstrated the potential for ERK to direct migration direction, it did not examine the coordination of mechanotransduction and ERK activity.

**Figure 4.**
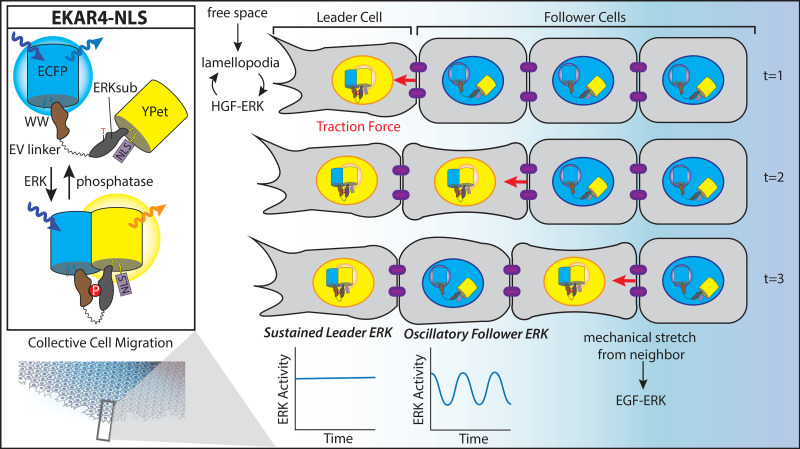
Cell-level ERK subpopulations in cell migration. Using a nuclear-localized ERK activity reporter with EV linker (EKARev-NLS) to investigate signaling heterogeneity among leader and follower cells during collective cell migration. With access to free space, leader cells undergo lamellipodial extension that facilitates a positive feedback loop to sustain ERK activity by increasing sensitivity to HGF. The mechanical stretch initiated by leader cells is propagated sequentially to follower cells, inducing EGF-based ERK activity that oscillates depending on neighboring mechanical stretch. Inspired by figures from Hino et al. [180,181].

Further work by Hino et al. [180] described a mechanochemical feedback system whereby mechanical stretch initiated by leader cells released from confinement induces ERK wave propagation through the sequential stretch and contraction of follower cells linked by cell–cell junctions. In this process, the leader cell pulls on the nearest follower cell, triggering ERK activity on the lead side while complementary RhoGTPase signaling along the opposite cell edge initiates contractile force to pull the next nearest follower cell. Thus, leader cells exhibit sustained EKARev-NLS FRET responses, while follower cells show lower FRET responses except when stretched by a neighboring cell. Later work by Hino et al. [181] examined epithelial cells released from mechanical confinement and found that cells with free space for lamellipodial extension acquire increased hepatocyte growth factor (HGF) sensitivity and that subsequent ERK signaling continues promoting lamellipodial extension into free space, thus harnessing a positive feedback loop to specify leader cell identity. While work on ERK signaling via epidermal growth factor (EGF) for collective cell migration had previously been the major focus, this new work revealed leader cells had HGF-dependent sustained ERK activation, whereas follower cells had EGF-dependent oscillatory ERK activity. Indeed, despite slowing overall population migration, EGFR inhibition alone only blocks follower-cell EKAR FRET responses; eliminating leader-cell EKAR responses required also removing HGF or targeting other downstream HGF-ERK signaling machinery. Taken together, these migration behaviors build a new model of collective cell migration where leader cells sustain ERK activation via HGF to maintain forward motion while follower cells sequentially stretch and contract to propagate ERK activity via EGF (Figure 4). These studies demonstrate the utility of biosensors to visualize cell–cell interactions and to complement other investigative methods involving advanced microscopy and image analysis to track distinct single-cell behaviors among cell populations.

## Conclusion

On a technical level, fluorescent biosensors represent highly adaptable tools to quantify signaling at different scales of spatial regulation, from the tissue-level all the way down to the nanoscale (Table 1). When pursuing biosensor applications, however, it's always important to consider how biosensor expression may impact signaling activity. For example, overexpressing chimeric biosensors that achieve subcellular targeting by incorporating full-length signaling proteins can alter the stoichiometry of native protein complexes and thus perturb signaling. More generic targeting strategies based on localization signals may lead to crowding at the target region or potentially overwhelm trafficking machinery, resulting in mislocalization of biosensors. Overexpressing a biosensor may also perturb signaling on its own, since biosensors often act as surrogate enzyme substrates or directly bind endogenous molecules, which can introduce buffering effects. Well-designed control experiments are therefore essential to rigorously confirm signaling phenomena and ensure proper interpretation of biosensor imaging studies. Meanwhile, biosensor engineering efforts to address these challenges moving forward remain a high priority. New designs such as FluoSTEPs, which enable biosensor targeting to endogenously expressed proteins, should be particularly helpful in this regard. More fundamentally, enhancing biosensor performance, such as by increasing sensitivity and signal-to-noise ratio, can enable robust reporting of biochemical activities with much lower biosensor expression, thereby reducing the risk of signaling perturbation.

**Table 1:** Overview of highlighted works within each scale of signaling, including the biosensors and other technology enabling biological discoveries

Scale	Section	Biosensor approaches	Key biological findings	Ref
Membranes and organelles	Lysosomal PI3K	C/Y-FRET PIP_3_ and PIP(3,4)P_2_ biosensor ‘InPAkt’ Single-fluorophore Akt biosensor ‘ExRai AktAR2' C/Y-FRET mTORC1 biosensor ‘TORCAR' Subcellular targeting via LAMP1	3-phosphoinositides accumulate at lysosomes via dynamin-mediated endocytosis Lysosomal 3-phosphoinositides promote growth factor-induced lysosomal Akt and mTORC1 activity	[48]
	Nuclear Akt/mTOR	Akt substrate-based tandem occupancy peptide sponge (Akt-STOPS) for local blockade of Akt signaling C/Y-FRET mTORC1 biosensor ‘TORCAR' C/Y-FRET Akt biosensor ‘AktAR2' Subcellular targeting via NLS or H2A	Nuclear mTORC1 activity depends on nuclear Akt during growth factor signaling but not nutrient signaling Nuclear Akt facilitates nuclear mTORC1 activity by regulating nuclear trafficking of Raptor	[58]
		C/Y-FRET mTORC1 biosensor ‘TORCAR' Subcellular targeting via NLS	Rheb regulates mTORC1 activity in the nucleus	[59]
	Nuclear, lysosomal, and mitochondrial AMPK	Single fluorophore AMPK biosensor ‘ExRai AMPKAR' Implementation in upstream kinase LKB1 and CAMKK2 knockout conditions Subcellular targeting via NLS, LAMP1, and DAKAP	AMPK is activated in the cytosol before entering the nucleus LKB1 drives cytosolic AMPK activity and rapid onset of AMPK lysosomal activity CAMKK2 drives maximal AMPK activity at multiple subcellular sites (cytosol, lysosomes, mitochondria)	[72]
	Endosomal GPCR-mediated ERK signaling	C/Y-FRET ERK biosensor ‘EKAR4' β2AR/Gαs perturbation tools: Nb80, Nb37, GsCT Subcellular targeting via NLS, KRAS, and FYVE	β2AR signaling does not induce plasma membrane ERK activity β-arrestin facilitates GPCR endocytosis; active endosomal Gαs turns on the ERK cascade on endosomes, ultimately inducing nuclear ERK signaling	[83]
Molecular assemblies	Endogenous PKA/cAMP in phase separation	Fluorescent sensors targeted to endogenous proteins (FluoSTEPs) — FRET-biosensors using spontaneous fragment complementation of a split sfGFP FRET donor to target endogenous proteins G/R-FRET cAMP biosensor ‘FluoSTEP-ICUE' G/R-FRET PKA biosensor ‘FluoSTEP-AKAR' Molecular targeting to endogenous type I PKA regulatory subunit via CRISPR/Cas9 tagging with FP_11_	RIα condensates contain high levels of cAMP and PKA activity compared with cytosolic pool of RIα cAMP stimulus induces formation of new RIα condensates that dynamically sequester cAMP and PKA	[94,96]
	cAMP regulatory fencing and compartmentation	C/Y-FRET cAMP biosensor ‘Epac1-camps' C/Y-FRET cAMP biosensor ‘ICUE4' C/Y-FRET PKA biosensor ‘AKAR4' Molecular targeting to PDEs with or without nanorulers to define cAMP accumulation and PKA activity within defined radius	PDEs actively degrade cAMP and block PKA activation to form nanoscale domains under low cAMP conditions cAMP undergoes buffered diffusion, possibly via RIα condensates, helping produce PDE nanodomains	[106]
	GPCR specificity in cAMP compartmentation	C/Y-FRET cAMP biosensor ‘Epac1-camps' C/Y-FRET PKA biosensor ‘AKAR4' Molecular targeting to GPCRs (GLP-1R or β2AR) with nanorulers to define cAMP accumulation and PKA activity within defined radius	Spatially confined cAMP gradients form locally around individual GPCRs upon agonist stimulation, dubbed receptor-associated independent cAMP nanodomains (RAINs) Physiological agonist concentrations induce cAMP elevations sufficient to activate PKA within 60 nm region surrounding a receptor	[108]
	AKAPs: PKA signaling islands	Fluorescence fluctuation increase by contact (FLINC) sensor technology compatible with photochromism-based super-resolution imaging Super-resolution compatible PKA biosensor ‘FLINC-AKAR1' Molecular targeting with KRAS and colocalization with AKAP79	PKA activity is confined within discrete nanodomains along the plasma membrane, which colocalize with nanoclusters of the PKA scaffold AKAP79	[119]
		Dronpa chromophore-removed FLINC (DrFLINC) sensor compatible with dual-color super-resolution imaging of DrFLINC sensor and Dronpa-labeled markers. PKA biosensor ‘DrFLINC-AKAR1' Molecular targeting with KRAS; colocalization with AKAP79, Actin, or Ca_v_1.2	Plasma membrane PKA activity nanodomains dynamically colocalize with AKAP79 clusters, actin-driven cell protrusions, and l-type calcium channels in live cells	[121]
Cell/tissue heterogeneity	Cell cycle tracking	Fluorescence ubiquitin cell cycle indicator ‘FUCCI' CDK1/2 translocation biosensor	Mitosis and cell cycle exit are driven by competing molecular ‘clocks'; cells can be driven to exit the cell cycle by delaying the mitotic clock at any point during interphase, not just in G1 Mitogens and CDK4/6 activity are required throughout interphase to maintain CDK2 activity for post-restriction point cells to proceed through mitosis	[155]
	AMPK & drivers of single cell metabolic status	C/Y-FRET AMPK biosensor ‘AMPKAR2' Green NAD+/NADH biosensor ‘Peredox' Akt translocation biosensor ‘Akt-KTR' Fluorescence ubiquitin cell cycle indicators ‘FUCCI' C/Y-FRET ATP biosensor ‘ATeam 1.03' C/Y-FRET ADP/ATP biosensor ‘PercevalHR'	Individual cells within a population can demonstrate oxidative phosphorylation (oxphos) dependence or independence. Cell metabolic status can be transiently inherited. Glycolytic capacity, cell cycle phase, and protein synthesis capacity affect oxphos dependence or independence of individual cells.	[160,161]
	ERK subpopulations in proliferation & apoptosis	C/Y-FRET ERK biosensor ‘EKARev' ± NLS Fluorescence ubiquitin cell cycle indicators ‘FUCCI'	Pulses of ERK activity are frequency modulated by growth factor receptor activity and amplitude modulated by upstream kinase MEK.	[166]
		C/Y-FRET ERK biosensor ‘EKARev' ± NLS Fluorescence ubiquitin cell cycle indicators ‘FUCCI'	Spatial radial ERK activity distributions (SPREADs) correlate with G2/M phase of the cell cycle.	[169]
		C/Y-FRET ERK biosensor ‘EKARev' ± NLS Fluorescence ubiquitin cell cycle indicators ‘FUCCI' Akt translocation biosensor ‘Akt-KTR'	Apoptotic cells initiate ERK and Akt activity waves as survival signals to neighboring cells to maintain cell barrier integrity.	[34]
	ERK subpopulations in cell migration	C/Y-FRET ERK biosensor ‘EKARev-NLS' Coupled with traction force microscopy	ERK activity waves precede myosin light change movement, inducing traction force generation in the opposite direction of ERK waves during migration.	[179]
		C/Y-FRET ERK biosensor ‘EKARev-NLS' Coupled with traction force microscopy	Mechanical stretch initiated by leader cells during migration induces ERK wave propagation through the sequential stretch and contraction of follower cells linked by cell–cell junctions.	[180]
		C/Y-FRET ERK biosensor ‘EKARev-NLS' Coupled with traction force microscopy	Leader cells sustain ERK activation via a positive feedback loop requiring HGF, whereas follower cells have sequential ERK activation requiring EGF.	[181]

The use of biosensors continues expanding from (1) limited signaling pathways to broader signaling pathways, (2) limited localizations to a broad spectrum of protein- and organelle-specific targeting, and (3) fewer cell/tissue types to broader adoption in a wide array of primary cells and animal tissues. For animal studies in particular, the poor *in vivo* performance of most traditional fluorescent biosensors, owing to the scattering and absorption of visible light by complex tissues, drives the ongoing need to adapt existing fluorescent biosensor designs to use red-shifted (e.g. far-red or near-infrared) fluorophores, as well as to increase compatibility with multiphoton microscopy methods, especially in neuroscience, for *in vivo*imaging. The modularity and utility of biosensors facilitates their integration with numerous technologies that are being used to improve our understanding of cell signaling in the 21st century. At the molecular scale, for example, genetically encoded fluorescent biosensors have been integrated with super-resolution microscopy techniques to illuminate nanoscale signaling events [119,121]. However, relatively few FPs are suitable for the development of biosensors compatible with super-resolution microscopy, where photobleaching risks are elevated or unique fluorophore properties such as photochromism are required. Increased development of biosensors to work with a wider array of super-resolution microscopy platforms in the future, driven by improvements in the engineering of FPs and other genetically encodable fluorescent tagging approaches, will thus be greatly desirable. At the level of membranes and organelles, as well as cell and tissue heterogeneity, fluorescent biosensors complement computer vision analyses aimed at tracking and quantifying single-cell behaviors. Indeed, the excellent temporal resolution of fluorescent biosensor data has contributed to several computational efforts to model cell signaling networks and compartments at numerous scales of spatial regulation [95,182–187]. As efforts to investigate more network properties in parallel are faced with spectral limitations inherent to multiplexing several biosensors in a single cell, we look forward to seeing more strategies that capitalize on analytical advancements to overcome this limitation, including catering biosensor design to specific analytical tasks, as was the case with massively multiplexed biosensors to quantify fourteen distinct biological activities simultaneously through deep-learning image analysis models [28].

On a biological level, fluorescent biosensor studies continue to reveal novel insights that broadly impact pharmaceutical and medical sciences. In this review, we highlighted recent applications of biosensors to describe aspects of spatial signaling regulation that are critical in numerous areas, including metabolism, cancer, and cell development. Just as the transcriptional, translational, and post-translational regulation of signaling elements are essential to study, understanding the spatial regulation of these components provides vital information to understand fundamental biological functions. Moreover, after biosensors identify signaling mechanisms in homeostatic contexts, they can be used to directly investigate contexts of disease-related deleterious signaling and to explore the mechanism of therapeutic interventions. Fluorescent biosensors thus enable us to go beyond simplified linear models of cell signaling to describe the intricate spatiotemporal regulation of signaling networks across scales that will illuminate 21st century biomedical research.

## Open Access

Open access for this article was enabled by the participation of University of California in an all-inclusive *Read & Publish* agreement with Portland Press and the Biochemical Society under a transformative agreement with UC.

## Abbreviations

ACs: adenylate cyclases
AKAPs: A-kinase anchoring proteins
AMP: adenosine monophosphate
AMPK: AMP-activated kinase
CAMKK2: calcium/calmodulin-dependent protein kinase kinase 2
DrFLINC: Dronpa chromophore-removed FLINC
EGF: epidermal growth factor
ER: endoplasmic reticulum
ERK: extracellular regulated kinase
FHA1: forkhead associated 1
FLINC: fluctuation increase by contact
FP: fluorescent protein
FRET: Förster resonance energy transfer
FUCCI: fluorescent ubiquitin cell cycle indicators
GFP: green fluorescent protein
GPCRs: G-protein coupled receptors
HGF: hepatocyte growth factor
ICUE: indicator of cAMP using Epac
KTRs: kinase translocation reporters
LAMP1: lysosome-associated membrane protein 1
LKB1: liver kinase B1
LLPS: liquid–liquid phase separation
NLS: nuclear localization sequence
OP: oxidative phosphorylation
PAABD: phospho-amino acid binding domain
PDEs: phosphodiesterases
PI3K: phosphatidylinositol 3-kinase
PIP3: phosphatidylinositol-3,4,5-triphosphate
PKA: protein kinase A
POI: protein of interest
RAINs: receptor-associated independent cAMP nanodomains
